# Books Reviewed

**Published:** 1866-11

**Authors:** 


### Books Reviewed.

Hamilton on Fractures and Dislocations.

The first appearance of this work was in the winter of 1859, since which time it has passed to its third edition. At the time of its first publication the English language did not contain another complete treatise upon Fractures and Dislocations, and the profession of this country have shown their appreciation of this work by the most substantial proofs; surgeons will always manifest their interest and confidence in it, especially so whenever deformities after injuries result in legal investigations. The profession, however, can never fully appreciate the value of this book, since it is such a bulwark of defence on both sides, preventing suits for mal-practice and justifying conditions of deformity which surgical skill cannot prevent.

In the preparation of this edition the cases and observations published since the date of the first have been studied, and valuable additions made both in the text and in the illustration of it. The chapter upon gun-shot wounds has been enlarged by the experiences of the late war, and the whole work brought up to the standard of practice at the present day, indeed is itself the standard, and by it must be estimated the results of surgical practice. Epiphyseal separations liable to be confounded with fractures are additionally illustrated by cuts selected from Gray's anatomy, indicating the centers of ossification and development of bone. The author describes these epiphyseal separations as quite distinct accidents, and as often requiring much longer time for repair than fracture of bone; he makes mention of such separation of the acromion process, upper and lower ends of the humerus, upper and lower ends of the femur, and trocanter major. These injuries are described carefully and their occurrence seems probable, but it does not appear that the proof is conclusive. He relates four cases in the femur supposed to be of this nature, and then says: "these four constitute the only examples at the upper end of the thigh, which I find reported, or of which I have any knowledge, and although there may be much reason to suppose that the diagnosis was correct in each instance, I cannot regard any one of them as actually proven; nor can I admit the accident as fully established, or the diagnostic signs as fully made out, until these important points have received the confirmation of at least one dissection." It seems probable that bones might separate more easily at their epiphyseal points than elsewhere, especially in young subjects, or in cases of delayed union, but we cannot now appreciate any circumstances or appearances which would enable the surgeon to determine the exact nature of the accident. If it should be settled that such accidents are common it still seems probable that nothing but dissection could determine its existence—differential diagnosis be- ween it and fracture will certainly be difficult if not impossible.

We are much gratified with the appearance of this edition, and though the former ones were supposed to be perfect, this is certainly an improvement upon the first and second. The distinguished author has earned his reputation, and his work will remain an enduring testimonial to his candor, zeal, intelligence and unhesitating avowal of the truth. He has spoken truthfully for the science and art of surgery, and at the same time for himself and the whole profession.

---

Flint on the Respiratory Organs.

It is now ten years since the first appearance of this work, which has beeu out of print for some time. During these ten years, the author has given special attention to the diagnosis of diseases affecting the respiratory organs, and has been constantly teaching at the bed-side and in didactic lectures the physical signs of these diseases. These facts would alone prove sufficient evidence of the value of the book, but the American profession are sufficiently well acquainted with this distinguished teacher and author to make any notice of his work other than the simple announcement of its appearance necessary.

The present edition has undergone considerable change, as compared with the first, though the general plan remains the same. The design of the work, is to present as plainly as consistent with full exposition, the physical signs of the diseases of the chest, which has been accomplished by "adopting a convenient classification, by pointing out distinctly their differential characters, and by the introduction of a few new names, which are in themselves description." Pitch of the resonance observed in percussion, and the pitch of the mucus, the subcriptant and criptant rales as indicative of the presence or absence of disease of the lung are considered much more fully than heretofore, as furnishing valuable diagnostic signs. Attention is also directed to a novel method of auscultatory percussion, which consists in "applying Cammann's stethoscope near to the open mouth of the patient, while percussion is made. In this way the amphoric and the cracked metal intonation may often be obtained in cases in which they are otherwise inappreciable."

The whole art of physical exploration is embraced in this work, together with the rational signs of disease, while the relative value of each is plainly indicated, so that it cannot be said that one set or group of symptoms have been considered to the exclusion of others. In its manner of arrangement and classifieation it is excellent, and in the accuracy and completeness of its teaching, perfect.

---

Nelligan on Diseases of the Skin.

In 1852 Dr. J. Moore Nelligan of Dublin, published a small work which he termed in his practice "A Concise Practical Treatise," diseases of the skin at that time having received comparatively little attention. This little work has passed to a second and enlarged Dublin edition and fifth American, thus showing the favor it has received from the profession of both countries. It still remains after being edited by Dr. T. W. Belcher, as originally produced, a concise, practical treatise upon diseases of the skin. The diseases of the skin are treated under the following general divisions: exanthemata, vesiculæ, pustulæ, squamæ, hypertrophiæ, hæmorrhagiæ, maculæ, cancroides, dermatrophytæ and syphilides. Chapters xiii and xiv are devoted to diseases of the appendages of the skin, and to therapeutics of diseases of the skin. It contains also biographical and general index of words and other matters. The work is, we believe, eminently adapted to the wants of the general practitioner, who has little time to consult the more voluminous works, and will prove also valuable to the student who desires condensation and accuracy.

---

A Hand-Book of Ophthalmic Surgery. By John Z. Laurence, F. R. C. S., M. B. (University London) and Robert C. Moon, House Surgeon to the Ophthalmic Hospital, Southwark. Philadelphia: Henry C. Lea, 1866.

The aim in writing this book, the author says, "is to bring the principles and practice of modern ophthalmic surgery within a small compass, to supply the wants of the busy practitioner, who may have neither time nor opportunity to read the innumerable contributions that ophthalmic surgery and science have received within the last fifteen years.

In describing symptoms, we have limited ourselves to those which are essential for the *recognition* of disease, in describing operations, etc., to those details which are essential for its *treatment*. At present, it matters little to the practitioner whether glaucoma depend on a hypersecretion of the fluids of the eye, or on a rheumatic state of the circulation, or on an obscure affection of the ciliary nerves, etc. What he chiefly wants is how to know glaucoma; and when he knows it, how to treat it."

We have quoted the language of the authors to show the true intention and scope of the work, and we believe that the design has been answered in a remarkable degree. The descriptions of disease are very plain and direct, the directions for using the ophthalmoscope are plain and easily understood. The representations of new instruments and of all the various methods of diagnosis by instrumental examination is complete and instructive. The whole work commends itself to most favorable consideration, and will be found one of the most attractive works upon the eye for the student and general practitiener.

Perhaps it is hoping too much to expect physicians engaged in the general practice of medicine to fully acquaint themselves with diseases of the eye and the various new methods of discovering them, but certain it is, physicions should pay more attention to this department of practice, time for which, might easily and properly enough be diverted from other and less attractive and less useful objects of professional investigation.

---

Elements of Medical Chemistry. By B. Howard Rand, M. D., Professor of Chemistry in the Jefferson Medical College. Philadelphia: T. Ellwood Zell & Co.

This work is designed chiefly for the use of students of medicine during their attendance upon lectures; but the practitioner will find comprised within its scope about all which he can desire to read, of medical chemistry and physics.

So far as time has permi tted examination we have been much pleased with the general arrangement and choice selection of topics, and we believe that no work of this character could be better adapted to the wants of medical students both before graduation and when the duties of active practice require completeness with brevity.

